# Harm perceptions across vaping product features: An on‐line cross‐sectional survey of adults who smoke and/or vape in the United Kingdom

**DOI:** 10.1111/add.16572

**Published:** 2024-06-05

**Authors:** Katherine East, Giang Vu, Tianze Sun, Kimberly D’Mello, Parvati Rose Perman‐Howe, Eve Taylor, Matilda Nottage, Leonie Sarah Brose, Deborah Robson, Ann McNeill

**Affiliations:** ^1^ National Addiction Centre, Institute of Psychiatry, Psychology and Neuroscience King's College London London UK; ^2^ School of Public Health Sciences University of Waterloo Waterloo Ontario Canada; ^3^ National Centre for Youth Substance Use Research University of Queensland St Lucia Queensland Australia; ^4^ Sheffield Centre for Health and Related Research University of Sheffield Sheffield UK

**Keywords:** Harm reduction, perceptions, risk, smoking, survey, vaping

## Abstract

**Background and aims:**

Vaping products are diverse with a wide variety of features, and popular products change rapidly. This study examined the features and types of vaping products that people who smoke and/or vape perceive contribute to the health harms of vaping.

**Design, setting and participants:**

This was a cross‐sectional survey co‐designed with adults who smoked/vaped and pre‐registered. An on‐line survey (November 2022) was used of a convenience sample of adults in the United Kingdom who smoked and/or vaped (*n* = 494).

**Measurements:**

As primary outcomes, respondents were asked to select any of 15 vaping product features they perceived might have any effect on the health harms of vaping (for each: selected, not selected). Independent variables were smoking/vaping status (smoke and vape; vape, formerly smoked; vape, never regularly smoked; smoke, do not currently vape); relative vaping harm perceptions [less harmful than smoking (accurate), equally/more harmful than smoking or do not know/refused (other)]. Binary logistic regressions were used to compare outcomes by current vaping/smoking status and relative harm perceptions, adjusting for age and sex.

**Findings:**

Most people (54.7%) selected between one and three features. The most frequently selected were nicotine concentration (62.2%) and amount of e‐liquid consumed (59.1%), followed by nicotine type (e.g. salt or freebase; 33.0%), source/purchase location (25.3%), flavours (24.7%), temperature to heat e‐liquid (21.1%), heat produced by device (20.9%), e‐liquid brand (20.9%), amount of emissions (18.6%), device type (e.g. disposable, pod, tank; 17.2%), material of tank (17.0%), power/wattage (13.0%), device brand (8.1%), device size (4.1%) and device weight (2.4%). Higher nicotine concentrations, more e‐liquid and salt (versus freebase) nicotine were perceived to confer greater harms. Disposables were perceived as slightly more harmful than reusable devices. There were few differences by current vaping/smoking status and between those with accurate (versus other) harm perceptions of vaping relative to smoking (*P* > 0.05 for most contrasts, adjusting for age and sex).

**Conclusions:**

Certain features and types of vaping products [higher nicotine concentrations, more e‐liquid consumed and salt (versus freebase) nicotine] were perceived to confer greater health harms among a sample of UK adults who smoked and/or vaped. Findings are consistent with pervasive misperceptions that nicotine is a major cause of harm, although e‐liquid volume is likely to contribute to harms.

## INTRODUCTION

E‐cigarettes (vaping products) typically contain nicotine, but not tobacco, and can help people to quit smoking [1, 2, 3]. While e‐cigarette use (vaping) carries some risks, current evidence suggests that it is substantially less harmful than smoking [2, 3, 4, 5]. However, public perceptions of the relative harm of vaping are often inaccurate [2, 6, 7, 8, 9, 10]. For example, in 2023, only one‐third of adults who smoked in Great Britain accurately perceived that vaping is less harmful than smoking, down from two‐thirds in 2013 [10]. Misperceptions of nicotine are also common [2, 6, 7, 8, 9, 11, 12]. In 2021, only 11% of adults who smoked in England knew that few to none of the health harms from smoking are due to nicotine [2].

Most research into vaping harm perceptions focuses upon relative harms compared with smoking, typically assessed through asking people whether they think vaping is less, equally or more harmful than smoking [2]. Vaping harm perceptions are also assessed through absolute harms, such as whether people think vaping is harmful or causes specific diseases and also whether nicotine is harmful or addictive [2]. However, the vaping product market is incredibly diverse, with various products and usage patterns, and little is known about people's perceptions of different vaping product features or types.

Different features or types of vaping products and the way that they are used may confer different risks to health [2, 3, 13]. For example, the emission of toxicants and their levels has been found to vary depending on vaping device and e‐liquid characteristics and how the product is operated [3, 14, 15]. Newer generations of disposable vaping products, currently the most popular type of vaping device in the United Kingdom [16, 17], typically contain synthetic coolants which may lead to different levels of toxicant exposure [18, 19]. Self‐reported data among youth also suggest that vaping disposables, compared with other devices, has been associated with shortness of breath, chest pain and phlegm [20]. There is limited evidence from animal and cell studies that buttery/creamy flavoured e‐liquids and e‐liquid containing cinnamaldehyde can alter cellular responses, but less so than tobacco smoke [2, 21]. Higher voltages, associated with higher battery outputs and higher temperatures to heat e‐liquids, can generate higher levels of carbonyl compounds [22]. Nicotine can be addictive and vaping with higher nicotine concentrations may have a greater potential for addiction than vaping with lower nicotine concentrations. However, vaping with lower nicotine concentrations could lead to people titrating their nicotine intake through inhaling larger aerosol volumes more deeply, for longer and more frequently, which may increase risk of harm through increasing exposure to aerosol constituents [13, 14, 15, 23]. Illicit vaping products pose a greater health risk because there is no assurance concerning the chemicals they contain and whether they are safe to be inhaled. In particular, vaping products that contain tetrahydrocannabinol (THC; i.e. cannabis), which are illicit in the United Kingdom, can contain contaminants that cause lung injuries [2]. However, these should not be confused with regulated nicotine vaping products.

In December 2021 we spoke to a group of adults who smoke and/or vape and asked them for their views on the harms of vaping and why [24]. It emerged that some people understand that the risks of vaping depend upon the vaping product and how it is used. For example, the group mentioned that consuming a greater amount of e‐liquid with a lower nicotine concentration might confer greater health harms, consistent with current evidence [2, 3, 13, 14, 15]. Some also believed that vaping products with fewer voluminous emissions conferred reduced harm, consistent with qualitative work on the heated tobacco product IQOS [25], and that certain brands were less harmful. To our knowledge, there has been no empirical research examining people's harm perceptions of the different features of vaping products and the way in which they are used.

Understanding people's harm perceptions of the different features of vaping products and how they are used is important to help design interventions to encourage adults who smoke to switch to vaping and allow people to make informed choices about the vaping products that they use. As above, there are pervasive misperceptions about vaping and nicotine harms [2, 6, 7, 8, 9, 10, 11, 12] and this can deter adults who smoke from switching to vaping and increase the likelihood of relapse to smoking once quit [2, 26]. Evidence also suggests that carefully designed interventions communicating that vaping is less harmful than smoking can correct vaping misperceptions [2, 27], but that interventions typically do not consider different vaping products or their features [2].

This study therefore examined the features and types of vaping products that people who smoke and/or vape perceive contribute to the health harms of vaping. Differences in perceptions by current smoking and vaping status, and between those with accurate (versus other) relative harm perceptions, were also examined because vaping perceptions among adults who continue to smoke and/or those who do not have accurate relative perceptions are the most important to address. Moreover, people who vape are generally more informed regarding vaping and nicotine [2, 26, 28]. This study was conducted in the United Kingdom in November 2022, after disposables became the most popular type of vaping device [16, 17].

## METHODS

### Public involvement and pre‐registration

This study emanated from discussions with adults who smoke and/or vape [24], and these same adults co‐designed the survey measures. Discussing research with people with living experience is important for two primary reasons. First, they have valuable first‐hand insights that can help progress the field [29]. Secondly, people have a right to be involved in research that affects them [29]. This study was pre‐registered (osf.io/kmze4) [30].

### Design and recruitment procedure

Data were collected in November 2022 using an on‐line cross‐sectional survey among adults aged 18+ in the United Kingdom who smoked and/or vaped. Participants were recruited using convenience sampling via Prolific Academic, a crowdsourcing platform with its own global pool of research participants that are verified using ID checks and vetted using attention, comprehension and honesty checks [31]. Participants are primarily recruited to Prolific via word of mouth (including via social media). For this study, participants from the United Kingdom only were eligible and were recruited through a survey link sent to eligible Prolific research participants. The target sample (500 participants) was obtained in under 24 hours.

The survey was designed and undertaken in Qualtrics, and screening questions were used to sample participants who currently smoked and/or vaped. First, Prolific Academic's internal screener set was applied to only include participants who reported that they: ‘Regularly use both tobacco products and e‐cigarettes’, ‘Previously smoked tobacco products. Now only use e‐cigarettes’, ‘Only ever used e‐cigarettes regularly (not tobacco products)’ and ‘Only use tobacco products’. Secondly, an additional screener was applied to only include participants who reported that they currently smoked and/or vaped at least monthly. Further details of the screening questions and routing are available in the study protocol [30]. The survey took approximately 5 minutes to complete.

Participants who completed the survey were reimbursed via Prolific Academic (55p for a median completion time of 3.35 minutes). A total of 566 participants took part, of whom 35 were ineligible (did not smoke and/or vape or did not report smoking/vaping status), 19 did not complete the survey and nine took part more than once. An additional eight had missing data on relative vaping harm perceptions and one on sex, so complete case analysis was used, which is acceptable when < 5% of the data are missing [32]. The final analytical sample comprised 494 participants.

### Measures

The survey measures including routing are available in the study protocol (osf.io/ac7yg) [30].

#### Primary outcomes

Participants were asked: ‘Which of the following features do you believe might have any effect on the health harms of vaping?’, with the following select‐all‐that‐apply response options (coded as selected versus did not select): (1) ‘amount of e‐liquid consumed’, (2) ‘amount of visible cloud (or plume) of emissions’, (3) ‘nicotine concentration’, (4) ‘nicotine type (e.g. salt or freebase)’, (5) ‘heat produced by the device (i.e. how hot to the touch the device is)’, (6) ‘temperature to heat the e‐liquid’, (7) ‘power/wattage of the device’, (8) ‘flavours (e.g. tobacco, menthol, fruit)’, (9) ‘type of device (e.g. disposable, pod, tank)’, (10) ‘material of the tank (e.g. glass, plastic)’, (11) ‘where the product is sourced or purchased (e.g. vape shop, on‐line)’, (12) ‘weight of the device’, (13) ‘size of the device’, (14) ‘brand of the device’ and (15) ‘brand of the e‐liquid’. Participants also had an opportunity to enter ‘other’ features, with a free‐text box. As per our pre‐registration, free‐text responses were explored, but none fitted within the above listed outcomes and there were too few responses to be grouped into a new, cohesive category (13 overall, six of which were about other ingredients; in our pre‐registration we specified at least 10 responses per outcome to model it) [30].

#### Secondary outcomes

Participants who selected at least one of the 15 features above were then subsequently asked: ‘Please indicate where along the scale you perceive the greatest harm to users’ health’, with different response options depending on the feature selected. For most features (e.g. ‘amount of e‐liquid consumed’), respondents could select a value between 0 (‘less e‐liquid consumed is more harmful’) to 10 (‘more e‐liquid consumed is more harmful’) or ‘do not know’; the mean score between 0 and 10 was calculated for these outcomes. For (8) ‘flavours’, (11) ‘source’, (14) ‘brand of the device’ and (15) ‘brand of the e‐liquid’, respondents were provided with a free‐text box to enter what they perceived to be the most harmful and least harmful. See the study protocol for the full list of measures [30].

#### Independent variables

##### Current smoking/vaping status

Participants were asked to respond to three questions about their current vaping, current smoking and former smoking experiences, with response options for each: daily, weekly, monthly, tried and never tried [30]. Four mutually exclusive groups were derived: people who (a) currently vape and currently smoke; (b) currently vape and formerly smoked; (c) currently vape but never regularly smoked; and (d) do not currently vape but currently smoke. Groups (a) and (d) were treated as the reference categories because misperceptions among adults who continue to smoke are the most important to assess.

##### Harm perceptions of vaping relative to smoking

Participants were asked: ‘Compared to smoking cigarettes, how harmful do you think using e‐cigarettes/vaping is?’, with response options: (a) ‘much less harmful’, (b) ‘somewhat less harmful’, (c) ‘equally harmful’, (d) ‘somewhat more harmful’, (e) ‘much more harmful’ and (f) ‘do not know’. Responses were coded into accurate (vaping is less harmful than smoking, a–b) versus other (vaping is equally/more harmful than smoking, or do not know/refused, c–f), consistent with prior work [7, 11, 26].

### Analyses

For each of the 15 primary outcomes, the proportion of participants who selected each outcome were first examined overall and by each level of the independent variables. Secondly, unadjusted and adjusted (for age group and sex) binary logistic regressions were used to compare vaping/smoking status groups on each of the outcomes. Thirdly, unadjusted and adjusted (for age group, sex and current smoking/vaping status) binary logistic regressions were used to compare participants with accurate versus other harm perceptions of vaping relative to smoking. Age group and sex were identified as covariates a priori [30], because vaping prevalence in England is higher among younger adults and males [33] and older adults are more likely to have inaccurate vaping and nicotine perceptions [28, 34].

For the secondary outcomes, the mean (and median, due to some distributions not being symmetrical) provided by participants on each of the features perceived to contribute to greater harms from vaping were reported overall and by each level of the independent variables. The proportion of participants who selected ‘do not know’ was also reported. Our pre‐registration [30] specified use of unadjusted and adjusted linear regressions to compare scores throughout current vaping/smoking status groups (adjusted for age group and sex) and by accurate versus other relative harm perceptions (adjusted age group, sex and current smoking/vaping status) and these are reported in the Supporting information, because of small sample sizes.

For all analyses, if any level of any independent variable had fewer than 10 participants providing a valid response to an outcome, that level was excluded from analyses via pairwise deletion. Pairwise deletion was used to maximise sample size across the overall study.

The data and code are available on‐line (osf.io/ka2zc) [30].

## RESULTS

### Sample characteristics

Table 1 shows the sample characteristics. Most were aged under 50 years, identified as White race/ethnicity, currently vape and either currently or formerly smoked. Approximately half were female and in full‐time employment. Most accurately perceived that vaping is less harmful than smoking, although this was not consistent across the current vaping/smoking subgroups (51.6% who currently only smoke accurately perceived that vaping is less harmful than smoking, compared with 80.4% who smoke and vape, 92.7% who vape only and formerly smoked and 80.0% who vape only and have never smoked).

**TABLE 1:** Sample characteristics (*n* = 494).

	*n*	%
**Age (years)**		
18–29	121	24.5
30–39	142	28.7
40–49	113	22.9
50–59	73	14.8
60–69	35	7.1
70+	10	2.0
**Sex**		
Female	251	50.8
Male	243	49.2
**Ethnicity/race**		
White	444	89.9
Asian	23	4.7
Mixed	16	3.2
Black	8	1.6
Other	3	0.6
**Employment status**		
Full‐time	240	48.6
Part‐time	76	15.4
Not in paid work (e.g. homemaker)	59	11.9
Unemployed (and job‐seeking)	33	6.7
Due to start a new job within next week	4	0.8
Other	82	16.5
**Current vaping/smoking status**		
Smoke and vape	225	45.6
Vape only, formerly smoked	164	33.2
Vape only, never smoked	10	2.0
Smoke only	95	19.2
**Harm perceptions of vaping relative to smoking**		
Accurate (vaping is less harmful than smoking)	390	78.9
Other (equally/more harmful, or do not know/refused)	104	21.1

### Features that are perceived to have any effect on vaping harms

**FIGURE 1:**
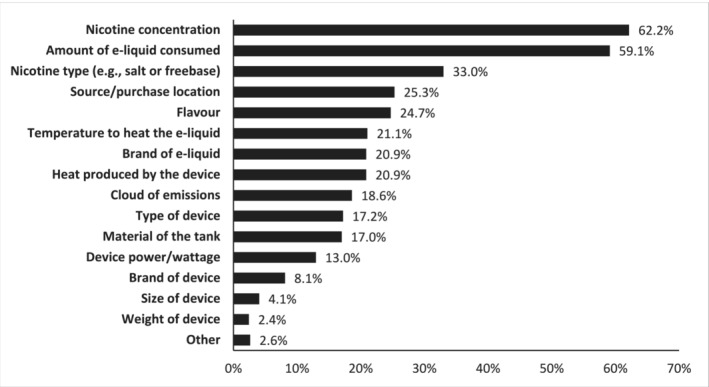
Proportion of sample who selected each feature that they perceived might have any effect on the health harms of vaping (*n* = 494).

Most people (*n* = 270, 54.7%) selected between one and three features of the 15 listed. The mean number of features selected was 3.5 [standard deviation (SD) = 2.4] and the median was 3.0. The most frequently selected features that were perceived to have any effect on the health harms of vaping were nicotine concentration (62.2%) and amount of e‐liquid consumed (59.1%), followed by nicotine type (e.g. salt or freebase; 33.0%), source/purchase location (25.3%), flavour (24.7%), temperature to heat e‐liquid (21.1%), heat produced by device (20.9%) and brand of e‐liquid (20.9%) (Figure 1). Remaining features were selected by fewer than one‐fifth of the sample (Figure 1).

#### Differences by current smoking/vaping status

Overall, most features perceived to contribute to the health harms of vaping were selected to a similar extent among adults regardless of their current vaping/smoking status (Table 2). However, there were some differences: ‘amount of e‐liquid consumed’ was selected more frequently by adults who vape and formerly smoked (65.0%) than adults who do both (54.4%), ‘flavours’ was selected more frequently by all other smoking/vaping subgroups (28.2–45.5%) compared with adults who do both (19.0%) and ‘material of the tank’ (e.g. glass, plastic) was selected more frequently among adults who smoke and do not vape (25.3%) than those who vape and formerly smoked (12.3%) (Table 2). In unadjusted analyses only, ‘type of device’ (e.g. pod, disposable) was selected more frequently by adults who vape and formerly smoked (22.7%) than adults who only smoke (11.6%) (Table 2). Nicotine concentration and amount of e‐liquid consumed were the most frequently selected across all smoking/vaping user groups.

**TABLE 2:** Associations between current vaping/smoking status groups and each primary outcome (feature perceived to contribute to the health harms of vaping; *n* = 494).

Vaping product features by current smoking/vaping	% (*n*)	Smoke and vape (a) as reference				Smoke, do not currently vape (d) as reference			
		OR (95% CI)	*P*	aOR^a^ (95% CI)	*P*	OR (95% CI)	*P*	aOR^a^ (95% CI)	*P*
**Nicotine concentration**									
(i) Smoke and vape	58.4 (132)	1.00		1.00		0.87 (0.53–1.42)	0.567	0.87 (0.53–1.45)	0.599
(ii) Vape, formerly smoked	66.3 (108)	1.36 (0.89–2.06)	0.150	1.31 (0.86–2.01)	0.207	1.18 (0.7–1.99)	0.544	1.15 (0.67–1.96)	0.614
(iii) Vape, never regularly smoked	72.7 (8)	2.82 (0.59–13.57)	0.196	2.53 (0.52–12.38)	0.252	2.44 (0.49–12.14)	0.276	2.21 (0.44–11.2)	0.339
(iv) Smoke, do not currently vape	62.1 (59)	1.15 (0.71–1.89)	0.567	1.15 (0.69–1.9)	0.599	1.00		1.00	
**Amount of e‐liquid consumed**									
(i) Smoke and vape	54.4 (123)	1.00		1.00		0.77 (0.47–1.25)	0.293	0.74 (0.45–1.21)	0.227
(ii) Vape, formerly smoked	65.0 (106)	**1.52 (1.00–2.29)**	**0.049**	**1.54 (1.01–2.34)**	**0.043**	1.17 (0.69–1.97)	0.564	1.13 (0.67–1.92)	0.641
(iii) Vape, never regularly smoked	45.5 (5)	0.83 (0.23–2.94)	0.772	0.74 (0.2–2.65)	0.640	0.64 (0.17–2.36)	0.500	0.54 (0.14–2.04)	0.366
(iv) Smoke, do not currently vape	61.1 (58)	1.3 (0.8–2.12)	0.293	1.36 (0.83–2.24)	0.227	1.00		1.00	
**Nicotine type (salt/freebase)**									
(i) Smoke and vape	33.2 (75)	1.00		1.00		1.14 (0.68–1.91)	0.624	1.05 (0.62–1.77)	0.869
(ii) Vape, formerly smoked	34.4 (56)	1.04 (0.68–1.59)	0.867	1.06 (0.69–1.63)	0.788	1.18 (0.69–2.03)	0.550	1.11 (0.64–1.93)	0.713
(iii) Vape, never regularly smoked	27.3 (3)	0.86 (0.22–3.41)	0.827	0.73 (0.18–2.93)	0.657	0.98 (0.24–4.04)	0.973	0.76 (0.18–3.21)	0.712
(iv) Smoke, do not currently vape	30.5 (29)	0.88 (0.52–1.47)	0.624	0.96 (0.56–1.62)	0.869	1.00		1.00	
**Source/purchase location**									
(i) Smoke and vape	23.0 (52)	1.00		1.00		0.68 (0.4–1.17)	0.165	0.69 (0.4–1.2)	0.187
(ii) Vape, formerly smoked	25.2 (41)	1.11 (0.69–1.77)	0.666	1.07 (0.66–1.72)	0.786	0.76 (0.43–1.33)	0.335	0.74 (0.42–1.31)	0.298
(iii) Vape, never regularly smoked	27.3 (3)	1.43 (0.36–5.71)	0.616	1.28 (0.31–5.22)	0.732	0.98 (0.24–4.04)	0.973	0.88 (0.21–3.76)	0.868
(iv) Smoke, do not currently vape	30.5 (29)	1.46 (0.86–2.5)	0.165	1.45 (0.84–2.5)	0.187	1.00		1.00	
**Flavours (e)g) tobacco, menthol, fruit)**									
(i) Smoke and vape	19.0 (43)	1.00		1.00		**0.57 (0.33–0.98)**	**0.043**	**0.53 (0.3–0.93)**	**0.027**
(ii) Vape, formerly smoked	28.2 (46)	**1.65 (1.03–2.66)**	**0.039**	**1.66 (1.02–2.69)**	**0.040**	0.93 (0.53–1.63)	0.807	0.88 (0.5–1.55)	0.646
(iii) Vape, never regularly smoked	45.5 (5)	**4.23 (1.17–15.27)**	**0.028**	**4.07 (1.11–14.97)**	**0.035**	2.39 (0.64–8.92)	0.194	2.15 (0.56–8.21)	0.264
(iv) Smoke, do not currently vape	29.5 (28)	**1.77 (1.02–3.07)**	**0.043**	**1.89 (1.08–3.34)**	**0.027**	1.00		1.00	
**Temperature to heat the e‐liquid**									
(i) Smoke and vape	19.5 (44)	1.00		1.00		0.76 (0.43–1.35)	0.351	0.68 (0.37–1.22)	0.192
(ii) Vape, formerly smoked	21.5 (35)	1.12 (0.68–1.84)	0.666	1.14 (0.69–1.88)	0.621	0.85 (0.47–1.55)	0.594	0.77 (0.42–1.42)	0.396
(iii) Vape, never regularly smoked	18.2 (2)	1.03 (0.21–5.01)	0.972	0.88 (0.18–4.38)	0.874	0.78 (0.16–3.95)	0.767	0.59 (0.11–3.09)	0.534
(iv) Smoke, do not currently vape	24.2 (23)	1.31 (0.74–2.33)	0.351	1.48 (0.82–2.67)	0.192	1.00		1.00	
**Heat produced by device**									
(i) Smoke and vape	19.5 (44)	1.00		1.00		0.81 (0.45–1.44)	0.467	0.64 (0.35–1.17)	0.150
(ii) Vape, formerly smoked	22.1 (36)	1.16 (0.71–1.9)	0.564	1.21 (0.73–2.01)	0.466	0.93 (0.51–1.71)	0.822	0.78 (0.41–1.45)	0.426
(iii) Vape, never regularly smoked	9.1 (1)	0.46 (0.06–3.7)	0.463	0.36 (0.04–2.98)	0.344	0.37 (0.04–3.07)	0.356	0.23 (0.03–1.98)	0.182
(iv) Smoke, do not currently vape	23.2 (22)	1.24 (0.69–2.21)	0.467	1.56 (0.85–2.85)	0.150	1.00		1.00	
**Brand of e‐liquid**									
(i) Smoke and vape	20.4 (46)	1.00		1.00		1.03 (0.57–1.87)	0.928	1.06 (0.57–1.94)	0.861
(ii) Vape, formerly smoked	21.5 (35)	1.06 (0.64–1.73)	0.830	1.02 (0.62–1.69)	0.932	1.09 (0.58–2.03)	0.798	1.08 (0.57–2.04)	0.815
(iii) Vape, never regularly smoked	27.3 (3)	1.67 (0.42–6.7)	0.471	1.71 (0.42–6.98)	0.456	1.71 (0.4–7.26)	0.464	1.8 (0.42–7.82)	0.431
(iv) Smoke, do not currently vape	20.0 (19)	0.97 (0.53–1.77)	0.928	0.95 (0.51–1.74)	0.861	1.00		1.00	
**Amount of visible cloud of emissions**									
(i) Smoke and vape	17.7 (40)	1.00		1.00		0.72 (0.4–1.29)	0.267	0.67 (0.37–1.22)	0.187
(ii) Vape, formerly smoked	17.2 (28)	0.95 (0.56–1.62)	0.857	0.95 (0.56–1.63)	0.861	0.68 (0.37–1.28)	0.233	0.64 (0.34–1.2)	0.165
(iii) Vape, never regularly smoked	18.2 (2)	1.16 (0.24–5.65)	0.858	0.98 (0.2–4.87)	0.984	0.83 (0.16–4.2)	0.821	0.66 (0.13–3.39)	0.615
(iv) Smoke, do not currently vape	23.2 (22)	1.39 (0.78–2.51)	0.267	1.5 (0.82–2.73)	0.187	1.00		1.00	
**Type of device (e)g) disposable, pod)**									
(i) Smoke and vape	15.5 (35)	1.00		1.00		1.41 (0.68–2.9)	0.356	1.26 (0.6–2.65)	0.543
(ii) Vape, formerly smoked	22.7 (37)	1.58 (0.95–2.64)	0.080	1.56 (0.92–2.64)	0.099	**2.22 (1.07–4.6)**	**0.031**	1.96 (0.93–4.13)	0.075
(iii) Vape, never regularly smoked	18.2 (2)	1.36 (0.28–6.66)	0.707	1.05 (0.2–5.37)	0.956	1.91 (0.36–10.16)	0.448	1.32 (0.24–7.39)	0.753
(iv) Smoke, do not currently vape	11.6 (11)	0.71 (0.34–1.47)	0.356	0.79 (0.38–1.67)	0.543	1.00		1.00	
**Material of tank (e)g) glass, plastic)**									
(i) Smoke and vape	16.8 (38)	1.00		1.00		0.6 (0.34–1.07)	0.085	0.59 (0.32–1.06)	0.078
(ii) Vape, formerly smoked	12.3 (20)	0.68 (0.38–1.22)	0.201	0.67 (0.37–1.2)	0.180	**0.41 (0.21–0.79)**	**0.008**	**0.39 (0.2–0.76)**	**0.006**
(iii) Vape, never regularly smoked	18.2 (2)	1.23 (0.25–6.02)	0.798	1.01 (0.2–5)	0.994	0.74 (0.15–3.73)	0.715	0.59 (0.11–3.04)	0.528
(iv) Smoke, do not currently vape	25.3 (24)	1.66 (0.93–2.97)	0.085	1.71 (0.94–3.1)	0.078	1.00		1.00	
**Power/wattage of device**									
(i) Smoke and vape	13.7 (31)	1.00		1.00		1.01 (0.5–2.02)	0.982	0.88 (0.43–1.8)	0.726
(ii) Vape, formerly smoked	11.7 (19)	0.82 (0.45–1.51)	0.524	0.8 (0.43–1.48)	0.469	0.83 (0.39–1.76)	0.621	0.7 (0.32–1.52)	0.366
(iii) Vape, never regularly smoked	9.1 (1)	0.7 (0.09–5.68)	0.735	0.55 (0.06–4.58)	0.576	0.7 (0.08–6)	0.746	0.48 (0.05–4.25)	0.509
(iv) Smoke, do not currently vape	13.7 (13)	0.99 (0.49–1.99)	0.982	1.14 (0.55–2.33)	0.726	1.00		1.00	
**Brand of device**									
(i) Smoke and vape	8.0 (18)	1.00		1.00		0.66 (0.3–1.47)	0.311	0.62 (0.27–1.4)	0.251
(ii) Vape, formerly smoked	6.1 (10)	0.75 (0.34–1.66)	0.475	0.72 (0.32–1.63)	0.436	0.5 (0.2–1.22)	0.125	0.45 (0.18–1.12)	0.087
(iii) Vape, never regularly smoked	9.1 (1)	1.28 (0.15–10.66)	0.821	1.08 (0.13–9.25)	0.945	0.85 (0.1–7.35)	0.881	0.67 (0.07–6.05)	0.720
(iv) Smoke, do not currently vape	11.6 (11)	1.51 (0.68–3.32)	0.311	1.61 (0.71–3.66)	0.251	1.00		1.00	
**Size of device**									
(i) Smoke and vape	3.5 (8)	1.00		1.00		0.46 (0.16–1.32)	0.149	0.5 (0.17–1.47)	0.207
(ii) Vape, formerly smoked	3.1 (5)	0.85 (0.27–2.66)	0.784	0.8 (0.25–2.52)	0.703	0.4 (0.12–1.28)	0.122	0.4 (0.12–1.33)	0.135
(iii) Vape, never regularly smoked	0.0 (0)	–	–	–	–	–	–	–	–
(iv) Smoke, do not currently vape	7.4 (7)	2.16 (0.76–6.13)	0.149	2 (0.68–5.85)	0.207	1.00		1.00	
**Weight of device**									
(i) Smoke and vape	1.3 (3)	1.00		1.00		0.31 (0.07–1.4)	0.127	0.35 (0.08–1.64)	0.183
(ii) Vape, formerly smoked	3.1 (5)	2.33 (0.55–9.88)	0.252	2.22 (0.52–9.49)	0.283	0.72 (0.19–2.73)	0.624	0.78 (0.2–3.04)	0.720
(iii) Vape, never regularly smoked	0.0 (0)	–	–	–	–	–	–	–	–
(iv) Smoke, do not currently vape	4.2 (4)	3.25 (0.71–14.82)	0.127	2.85 (0.61–13.28)	0.183	1.00		1.00	

*Note*: Excluded via pairwise deletion due to 0 responses. Bolded values are statistically significant (*P* < 0.05).

^a^ Adjusted for age group (18–29, 30–39, 40–49, 50+) and sex (male, female).

#### Differences by vaping harm perceptions

Overall, most features perceived to contribute to the health harms of vaping were selected to a similar extent among adults who accurately perceived vaping as less harmful than smoking compared with adults who had inaccurate perceptions or did not know (Table 3). However, there were some differences: compared with adults who had accurate relative vaping perceptions, ‘type of device’ (in adjusted analyses, 23.1 versus 15.6%) and ‘size of device’ (in unadjusted analyses; 8.7 versus 2.8%) were selected more frequently by adults who had inaccurate perceptions or did not know (Table 3).

**TABLE 3:** Associations between accurately perceiving vaping as less harmful than smoking and each primary outcome (feature perceived to contribute to the health harms of vaping; *n* = 494).

Vaping product features by harm perceptions	% (*n*)	Unadjusted		Adjusted^a^	
		OR (95% CI)	*P*	aOR^a^ (95% CI)	*P*
**Nicotine concentration**					
Accurate (vaping less harmful than smoking, ref.)	62.6 (244)	1.00		1.00	
Other^b^	60.6 (63)	0.92 (0.59–1.43)	0.710	0.93 (0.57–1.52)	0.783
**Amount of e‐liquid consumed**					
Accurate (vaping less harmful than smoking, ref.)	59.5 (232)	1.00		1.00	
Other^b^	57.7 (60)	0.93 (0.6–1.44)	0.741	0.94 (0.58–1.52)	0.802
**Nicotine type (e.g. salt or freebase)**					
Accurate (vaping less harmful than smoking, ref.)	32.1 (125)	1.00		1.00	
Other^b^	36.5 (38)	1.22 (0.78–1.92)	0.388	1.33 (0.81–2.2)	0.257
**Source/purchase location**					
Accurate (vaping less harmful than smoking, ref.)	24.4 (95)	1.00		1.00	
Other^b^	28.8 (30)	1.26 (0.78–2.04)	0.350	1.12 (0.66–1.91)	0.677
**Flavours (e.g. tobacco, menthol, fruit)**					
Accurate (vaping less harmful than smoking, ref.)	24.1 (94)	1.00		1.00	
Other^b^	26.9 (28)	1.16 (0.71–1.9)	0.554	1.15 (0.67–1.99)	0.610
**Temperature to heat the e‐liquid**					
Accurate (vaping less harmful than smoking, ref.)	21.0 (82)	1.00		1.00	
Other^b^	21.2 (22)	1.01 (0.59–1.71)	0.977	0.89 (0.5–1.59)	0.694
**Heat produced by device**					
Accurate (vaping less harmful than smoking, ref.)	19.7 (77)	1.00		1.00	
Other^b^	25.0 (26)	1.35 (0.81–2.25)	0.242	1.29 (0.73–2.28)	0.381
**Brand of e‐liquid**					
Accurate (vaping less harmful than smoking, ref.)	19.5 (76)	1.00		1.00	
Other^b^	26.0 (27)	1.45 (0.87–2.4)	0.150	1.56 (0.9–2.73)	0.115
**Amount of visible cloud/plume of emissions**					
Accurate (vaping less harmful than smoking, ref.)	17.4 (68)	1.00		1.00	
Other^b^	23.1 (24)	1.42 (0.84–2.4)	0.191	1.24 (0.7–2.22)	0.460
**Type of device (e.g. disposable, pod, tank)**					
Accurate (vaping less harmful than smoking, ref.)	**15.6 (61)**	1.00		1.00	
Other^b^	**23.1 (24)**	1.62 (0.95–2.75)	0.076	**2.04 (1.12–3.75)**	**0.021**
**Material of tank (e.g. glass, plastic)**					
Accurate (vaping less harmful than smoking, ref.)	16.4 (64)	1.00		1.00	
Other^b^	19.2 (20)	1.21 (0.7–2.12)	0.497	0.82 (0.45–1.52)	0.537
**Power/wattage of device**					
Accurate (vaping less harmful than smoking, ref.)	11.8 (46)	1.00		1.00	
Other^b^	17.3 (18)	1.57 (0.86–2.83)	0.139	1.48 (0.77–2.86)	0.244
**Brand of device**					
Accurate (vaping less harmful than smoking, ref.)	7.4 (29)	1.00		1.00	
Other^b^	10.6 (11)	1.47 (0.71–3.06)	0.299	1.25 (0.56–2.81)	0.583
**Size of device**					
Accurate (vaping less harmful than smoking, ref.)	**2.8 (11)**	1.00		1.00	
Other^b^	**8.7 (9)**	**3.26 (1.31–8.1)**	**0.011**	2.58 (0.93–7.17)	0.069
**Weight of device**					
Accurate (vaping less harmful than smoking, ref.)	1.8 (7)	1.00		1.00	
Other^b^	4.8 (5)	2.76 (0.86–8.89)	0.088	2.88 (0.73–11.33)	0.131

*Note*: Bolded values are statistically significant (*P* < 0.05).

^a^ Adjusted for age group (18–29, 30–39, 40–49, 50+), sex (male, female) and current smoking/vaping status (smoke and vape, vape and formerly smoked, vape and never regularly smoked, smoke and do not currently vape).

^b^ ‘Other’ comprises perceptions that vaping is equally/more harmful than smoking, or do not know/refused.

### Degree to which features are perceived to affect vaping harms

The scales for perceived harms of individual features ranged from 0 to 10. Average scores were greater than 5 across most features, indicating that participants generally perceived greater harms from: 2% (versus 0%) nicotine concentrations, more e‐liquid consumed, salt‐based (versus freebase) nicotine, higher temperature to heat the e‐liquid, more heat produced by the device, greater amount of emissions, plastic (versus glass) tank, higher (versus lower) powered devices and larger devices and heavier devices (Table 4). The two device‐type outcomes (pod versus tank; disposable versus reusable) had mean scores of 5 and 4 (median scores of 4.0 and 4.5), respectively, suggesting that participants generally perceived little difference in harms between tanks compared with pods but perceived disposables as slightly more harmful than reusable devices overall (Table 4). ‘Do not know’ responses ranged from 46.0% for nicotine type (*n* = 75 of *n* = 163) to 3.6% for nicotine concentration (*n* = 11 of *n* = 307; Table 4).

**TABLE 4:** Descriptive statistics for each secondary outcome (degree to which each feature is perceived to contribute to greater harms of vaping) overall and by current smoking/vaping status and harm perceptions.

		Nicotine concentration^a^ (2% versus 0%)	Amount of e‐liquid consumed^b^	Nicotine type^c^ (salt versus freebase)	Temperature to heat the e‐liquid^d^	Heat produced by the device^e^	Amount of visible cloud of emissions^f^	Device type^g^ (tank versus pod)	Device type^h^ (reusable versus disposable)	Material of tank^i^ (plastic versus glass)	Device power^j^	Size of device^k^	Weight of device^l^
**Overall**	*n*	307	292	163	104	103	92	85	85	84	64	20	12
**Do not know**	*n*	11	10	75	9	11	9	35	21	8	5	4	3
**Scores (excluding do not know)**	*n*	296	282	88	95	92	83	50	64	76	59	16	9
	Mean (SD)	8.46 (1.67)	8.01 (1.79)	6.52 (2.37)	7.58 (1.89)	7.33 (1.85)	7.54 (1.68)	5.00 (2.28)	4.34 (3.03)	7.99 (2.19)	7.32 (2.33)	7.06 (1.65)	6.22 (2.49)
	Median	9.0	8.0	7.0	8.0	7.0	8.0	5.0	4.5	8.5	7.0	7.0	6.0
**By current smoking/vaping:**													
(i) Smoke and vape	*n*	129	119	45	41	41	38	22	27	35	29	8	3
	Mean (SD)	8.44 (1.61)	7.86 (1.81)	6.18 (2.56)	8.02 (1.54)	7.12 (2.03)	7.37 (1.60)	5.09 (2.49)	4.44 (3.18)	8.17 (2.22)	7.10 (2.30)	7.00 (1.93)	7.67 (2.52)
	Median	9.0	8.0	6.0	8.0	7.0	7.5	5.0	4.0	9.0	7.0	7.0	8.0
(ii) Vape, formerly smoked	*n*	105	102	31	32	32	26	25	30	18	17	4	4
	Mean (SD)	8.47 (1.73)	8.06 (1.87)	6.81 (2.12)	7.16 (1.94)	7.41 (1.78)	7.42 (1.86)	4.96 (2.23)	4.03 (2.93)	8.22 (1.66)	7.29 (2.57)	7.75 (1.71)	5.00 (2.83)
	Median	9.0	8.0	7.0	7.5	7.5	8.0	5.0	4.0	8.5	7.0	7.5	4.0
(iii) Vape, never regularly smoked	*n*	8	5	1	2	1	2	2	2	2	1	0	0
	Mean (SD)	7.38 (1.41)	7.60 (1.52)	6 (–)	7 (0)	7 (–)	8 (2.83)	4.50 (2.12)	5.50 (3.54)	7.00 (1.41)	6 (–)	– (–)	– (–)
	Median	7.0	7.0	6.0	7.0	7.0	8.0	4.5	5.5	7.0	6.0	–	–
(iv) Smoke, do not currently vape	*n*	54	56	11	20	18	17	1	5	21	12	4	2
	Mean (SD)	8.63 (1.71)	8.29 (1.64)	7.18 (2.32)	7.40 (2.39)	7.67 (1.64)	8.06 (1.52)	5 (–)	5.20 (3.35)	7.57 (2.60)	8 (2.17)	6.5 (1.00)	6.50 (0.71)
	Median	9.0	8.0	7.0	8.0	8.0	8.0	5.0	5.0	8.0	8.0	6.0	6.5
**By relative harm perceptions:**													
(i) Accurate (vaping is less harmful than smoking)	*n*	238	225	69	76	68	64	36	46	58	41	9	5
	Mean (SD)	8.45 (1.65)	7.98 (1.78)	6.29 (2.14)	7.57 (1.65)	7.16 (1.92)	7.27 (1.54)	4.81 (2.21)	4.22 (3.06)	8.02 (2.08)	7.37 (2.15)	7.00 (1.66)	5.60 (2.79)
	Median	9.0	8.0	6.0	7.0	7.0	7.0	5.0	4.0	8.0	8.0	7.0	5.0
(ii) Other^m^	*n*	58	58	58	58	58	58	58	58	58	58	58	58
	Mean (SD)	8.48 (1.73)	8.12 (1.85)	7.37 (3.00)	7.63 (2.69)	7.79 (1.59)	8.47 (1.84)	5.50 (2.44)	4.67 (3.01)	7.89 (2.56)	7.22 (2.76)	7.14 (1.77)	7.00 (2.16)
	Median	9.0	8.0	8.0	9.0	7.5	9.0	5.0	5.0	9.0	7.0	7.0	6.5

*Note*: Unreportable due to low sample size. The full range of responses are shown in Supporting information, Table S1.

^a^ 0 = 0% nicotine (or 0 mg/ml) is more harmful; 10 = 2% nicotine (or 20 mg/ml) is more harmful.

^b^ 0 = less e‐liquid consumed is more harmful; 10 = more e‐liquid consumed is more harmful.

^c^ 0 = freebase is more harmful; 10 = salt is more harmful.

^d^ 0 = lower temperature is more harmful; 10 = higher temperature is more harmful.

^e^ 0 = less heat is more harmful; 10 = more heat is more harmful.

^f^ 0 = small visible cloud of emissions is more harmful; 10 = large visible cloud of emissions is more harmful.

^g^ 0 = pod device is more harmful; 10 = tank device is more harmful.

^h^ 0 = disposable device is more harmful; 10 = reusable device is more harmful.

^i^ 0 = glass tank is more harmful; 10 = plastic tank is more harmful.

^j^ 0 = low device power is more harmful; 10 = high device power is more harmful.

^k^ 0 = smaller device is more harmful; 10 = larger device is more harmful.

^l^ 0 = lighter device is more harmful; 10 = heavier device is more harmful.

^m^ 0 = vaping is equally/more harmful than smoking or do not know/refused.

## DISCUSSION

Among a convenience sample of adults who smoked and/or vaped in the United Kingdom in November 2022, certain features of vaping products were perceived to confer greater health harms. Specifically, nicotine concentration, the amount of e‐liquid consumed and nicotine type (salt/freebase) were the three most selected features that might have any effect on the health harms of vaping, with higher nicotine concentrations, more e‐liquid consumed and salt (versus freebase) nicotine perceived to confer greater harms. Other features, such as source, flavour, temperature, brand and power/wattage, were selected by fewer than one‐third of the sample. Device type was selected by 17%, and disposables were perceived as slightly more harmful than reusable devices. Perceptions were generally similar among adults who only vaped, only smoked and did both, as well as among those with accurate and other perceptions of vaping relative to smoking.

Findings are consistent with pervasive misperceptions in the general population that nicotine is a major contributor of harm [2, 6, 7, 8, 9, 11, 12]. Nicotine can have acute cardiovascular effects [35], and the survey item used in this study assessed perceptions of ‘any effect’ of vaping product features on vaping harms, which may potentially explain why nicotine was most commonly selected. Isolating the effects of inhaled nicotine on health risks from vaping is complex, and reviews of the literature have not been able to draw firm conclusions [2, 3, 13]. However, overall health risks of nicotine *per se* are small [35, 36], reviews of nicotine replacement therapies (NRT) have not found evidence of long‐term health effects [37] and the evidence is clear that it is not the nicotine that kills people who smoke. There is some evidence that vaping with lower nicotine concentrations could confer greater health risks if people titrate their nicotine intake through inhaling more aerosol [13, 15, 23].

More than half of our sample perceived that the amount of e‐liquid consumed might have any effect on the health harms of vaping. This is in line with current evidence [13, 15, 23] and our discussions with adults who smoke and/or vape [24]. Some also perceived that source/purchase location (25%), flavours (25%), device power (13%) and temperature to heat the e‐liquid (21%) might affect vaping harms, and there is some evidence that these are correct perceptions [2, 21, 22]. The finding that disposables were generally perceived to be slightly more harmful than reusable devices is consistent with some evidence from toxicology and self‐report data [18, 19, 20]. However, more data are required regarding the health harms of newer disposable vaping products on the market. The rapid increase in use of disposables among younger people [16, 17], and widespread media coverage of their use and environmental impacts, may also have contributed to negative overall views of disposable vapes.

Perceptions were generally similar among adults who only vaped, only smoked and did both and between those who had overall accurate (versus other) perceptions of vaping relative to smoking. These findings are surprising because people who vape are generally more informed about vaping and nicotine [2, 26, 28], and one would also expect people with accurate relative perceptions to be better informed. However, the accurate perception that the amount of e‐liquid consumed contributes to the health harms of vaping was more common among adults who vape and had quit smoking than adults who both smoked and vaped, suggesting that people who have switched completely may be better informed about vaping in some ways. Vaping perceptions among adults who continue to smoke are the most important to address, because inaccurate perceptions could deter switching to vaping and also increase the risk of relapse once quit [2, 26].

It is important to consider how people define health harms. Qualitative research suggests that some people include dependence or addiction when discussing harm [25, 38], and the addictiveness of nicotine depends upon multiple factors of the product and how it is used. Further work is required to understand the extent to which people perceive nicotine concentration contributes to vaping dependence and what ‘harms’ people consider in relation to vaping. Further research is also required on harms across the broad range of vaping products.

Findings highlight the need for evidence‐based interventions that educate adults who smoke regarding vaping and nicotine. The most important messages remain that using regulated, legal e‐cigarettes is substantially less harmful than smoking, and that it is not the nicotine that kills people who smoke. There should also be better education concerning nicotine concentrations and how this might impact use; specifically, that vaping with higher nicotine concentrations could lead to inhaling less aerosol less frequently and less deeply, but could invoke greater dependence [13, 14, 15, 23]. Such information is crucial, because inadequate knowledge regarding nicotine heath harms can impact informed decision‐making about quitting smoking [12]. Messages could also inform people to ensure that their vaping devices are well maintained and do not overheat, because these could increase health risks. More broadly, additional interventions and policies are required to encourage quitting smoking, because harm perceptions are only one contributor to behaviour change [39].

This study has limitations. First, data were not nationally representative—the convenience sample comprised 494 adults who smoked and/or vaped in the United Kingdom and, unlike the population of adults who smoke in the United Kingdom [2, 8, 10, 26, 28, 40], most accurately perceived that vaping is less harmful than smoking. Perceptions of harm from different vaping product features may therefore be greater overall, and potentially less accurate, among adults who smoke, and the wider UK population, compared to those in this study. Secondly, the item list was not comprehensive—other features that can influence vaping risks, such as coils and propylene glycol (PG) to vegetable glycerine (VG) ratio [41, 42, 43], were not considered. Thirdly, sample sizes for some planned analyses were small, resulting in wide confidence intervals and therefore limited confidence in contrasting perceptions between adults who smoked, vaped and did both. However, strengths include that this is the first study, to our knowledge, to assess specific features that people might perceive contribute to the health harms of vaping, and data provide new insights into vaping harm perceptions. The survey was co‐designed with a public involvement group of adults who smoke/vape, and so tailored to our target population, and considered the views of people who will be most impacted.

In conclusion, findings are consistent with pervasive misperceptions among the general population that nicotine is a major cause of harm. Interventions should emphasize that using regulated, legal e‐cigarettes is substantially less harmful than smoking and that nicotine, while addictive, is not the primary lethal component of cigarette smoke. Further research is required regarding harms throughout the broad range of vaping products and features using nationally representative data, and findings communicated to the public so that people can make informed decisions about the products that they use.

## AUTHOR CONTRIBUTIONS

**Katherine East:** Conceptualization (lead); data curation (lead); formal analysis (lead); funding acquisition (lead); investigation (lead); methodology (lead); project administration (lead); software (lead); validation (equal); visualization (lead); writing—original draft (lead); writing—review and editing (lead). **Giang Vu:** Data curation (equal); formal analysis (equal); writing—review and editing (equal). **Tianze Sun:** Data curation (equal); formal analysis (equal); writing—review and editing (equal). **Kimberly D'Mello:** Writing—review and editing (equal). **Parvati Rose Perman‐Howe:** Investigation (supporting); methodology (supporting); software (supporting); writing—review and editing (equal). **Eve Taylor:** Investigation (supporting); writing—review and editing (equal). **Matilda Nottage:** Investigation (supporting); writing—review and editing (equal). **Leonie Sarah Brose:** Investigation (supporting); writing—review and editing (equal). **Deborah Robson:** Investigation (supporting); writing—review and editing (equal). **Ann McNeill:** Investigation (supporting); writing—review and editing (equal).

## DECLARATION OF INTERESTS

P.P.H., L.B., D.R. and A.M. are members of SPECTRUM. All other authors have no conflicts of interest to declare.

## Supporting information


**Table S1.** Distribution of responses for the degree to which each feature is perceived to affect vaping harms.
**Table S2.** Linear regressions assessing the associations between vaping/smoking status groups and scores for the degree to which type of device (pod versus tank; disposable versus reusable) is perceived to contribute to the health harms of vaping.
**Table S3.** Linear regressions assessing the associations between vaping harm perceptions and scores for the degree to which type of device (pod versus tank; disposable versus reusable) is perceived to contribute to the health harms of vaping.
**Table S4.** Logistic regressions assessing the associations between vaping/smoking status groups high scores (>5) for the degree to which each feature is perceived to contribute to the health harms of vaping.
**Table S5.** Logistic regressions assessing the associations between vaping harm perceptions and high scores (>5) for the degree to which each feature is perceived to contribute to the health harms of vaping.

**Data S1.** Supplementary Information.

## Data Availability

The data and code are available on‐line (osf.io/ka2zc).
