# Supplementary material for: Harm perceptions across vaping product features: An on‐line cross‐sectional survey of adults who smoke and/or vape in the United Kingdom
Source: Addiction. 2024 Jun 5;120(3):524–38. doi: 10.1111/add.16572 (PMC11813726; doi:10.1111/add.16572)
Supplement: Supplementary file 1 — Table S1. Distribution of responses for the degree to which each feature is perceived to affect vaping harms. Table S2. Linear regressions assessing the associations between vaping/smoking status groups and scores for the degree to which type of device (pod versus tank; disposable versus reusable) is perceived to contribute to the health harms of vaping. Table S3. Linear regressions assessing the associations between vaping harm perceptions and scores for the degree to which type of device (pod versus tank; disposable versus reusable) is perceived to contribute to the health harms of vaping. Table S4. Logistic regressions assessing the associations between vaping/smoking status groups high scores (>5) for the degree to which each feature is perceived to contribute to the health harms of vaping. Table S5. Logistic regressions assessing the associations between vaping harm perceptions and high scores (>5) for the degree to which each feature is perceived to contribute to the health harms of vaping. [file ADD-120-524-s001.docx]

SUPPLEMENTARY TABLES AND FIGURES

**Supplementary Table 1. Distribution of responses for the degree to which each feature is perceived to affect vaping harms.**

|  | **Nicotine concentration^1^ (2% vs. 0%)** | **Amount of e-liquid consumed^2^** | **Nicotine type^3^ (salt vs. freebase)** | **Temperature to heat the e-liquid^4^** | **Heat produced by the device^5^** | **Amount of visible cloud of emissions^6^** | **Device type^7^ (tank vs. pod)** | **Device type^8^ (reusable vs. disposable.)** | **Material of tank (plastic vs. glass)^9^** | **Device power^10^** | **Size of device^11^** | **Weight of device^12^** |
| --- | --- | --- | --- | --- | --- | --- | --- | --- | --- | --- | --- | --- |
| **N** | 307 | 292 | 163 | 104 | 103 | 92 | 85 | 85 | 84 | 64 | 20 | 12 |
| **Distribution of responses (n)** |  |  |  |  |  |  |  |  |  |  |  |  |
| **0** | 1 | 2 | 1 | 0 | 0 | 0 | 1 | 10 | 1 | 1 | 0 | 0 |
| **1** | 0 | 1 | 1 | 2 | 0 | 0 | 0 | 3 | 1 | 0 | 0 | 0 |
| **2** | 1 | 2 | 5 | 1 | 1 | 0 | 7 | 7 | 2 | 2 | 0 | 0 |
| **3** | 4 | 4 | 2 | 0 | 4 | 0 | 6 | 8 | 0 | 2 | 0 | 2 |
| **4** | 0 | 3 | 5 | 1 | 2 | 6 | 7 | 4 | 1 | 3 | 0 | 0 |
| **5** | 4 | 5 | 16 | 4 | 6 | 3 | 9 | 9 | 1 | 2 | 3 | 2 |
| **6** | 25 | 19 | 12 | 15 | 11 | 12 | 9 | 8 | 9 | 4 | 4 | 1 |
| **7** | 43 | 61 | 14 | 22 | 25 | 18 | 2 | 2 | 9 | 16 | 3 | 1 |
| **9** | 46 | 44 | 9 | 12 | 11 | 13 | 3 | 4 | 16 | 6 | 1 | 1 |
| **10** | 114 | 70 | 11 | 18 | 13 | 12 | 1 | 3 | 22 | 13 | 2 | 1 |
| **Don't know** | 11 | 10 | 75 | 9 | 11 | 9 | 35 | 21 | 8 | 5 | 4 | 3 |

^1^ 0 = 0% nicotine (or 0mg/mL) is more harmful; 10 = 2% nicotine (or 20mg/mL) is more harmful.

^2^ 0 = less e-liquid consumed is more harmful; 10 = more e-liquid consumed is more harmful.

^3^ 0 = freebase is more harmful; 10 = salt is more harmful.

^4^ 0 = lower temperature is more harmful; 10 = higher temperature is more harmful.

^5^ 0 = less heat is more harmful; 10 = more heat is more harmful.

^6^ 0 = small visible cloud of emissions is more harmful; 10 = large visible cloud of emissions is more harmful.

^7^ 0 = pod device is more harmful; 10 = tank device is more harmful.

^8^ 0 = disposable device is more harmful; 10 = reusable device is more harmful.

^9^ 0 = glass tank is more harmful; 10 = plastic tank is more harmful.

^10^ 0 = low device power is more harmful; 10 = high device power is more harmful.

^11^ 0 = smaller device is more harmful; 10 = larger device is more harmful.

^12^ 0 = lighter device is more harmful; 10 = heavier device is more harmful.

Secondary outcomes: Degree to which features are perceived to affect vaping harms

Coding and analyses of secondary outcomes

Participants who selected at least one of the 15 features above were then subsequently asked “Please indicate where along the scale you perceive the greatest harm to users’ health” with different response options depending on the feature selected. For most features (e.g., “amount of e-liquid consumed”), respondents could select a value between 0 (‘less e-liquid consumed is more harmful’) to 10 (‘more e-liquid consumed is more harmful’) or ‘don’t know’; the mean score between 0 and 10 was calculated for these outcomes. For 8) ‘flavours’, 11) ‘source’, 14) ‘brand of the device’ and 15) ‘brand of the e-liquid’, respondents were provided with a free-text box to write in what they perceived to be the most harmful and least harmful. See the study protocol for the full list of measures (osf.io/kmze4).

Our pre-registration (osf.io/kmze4) specified use of unadjusted and adjusted linear regressions to compare average scores across vaping/smoking status user groups (adjusted for age group and sex) and by accurate vs. other relative harm perceptions (adjusted age group, sex, and smoking/vaping status) or, where assumptions were violated, to dichotomise scores and use binary logistic regressions. The assumption of normally distributed residuals was violated for all secondary outcomes, except device type when log-transformed, and so, for consistency across outcomes, all scores were dichotomised with scores between 0 and 5 coded as ‘less/equal harm’ and scores between 6 and 10 as ‘more harm’. Linear regression was also reported for device type (log-transformed) as per the pre-registration (osf.io/kmze4). These analyses are reported below for consistency with the pre-registration but are not reported in the manuscript due to low sample sizes and therefore lack of confidence in the findings.

Findings

Except the two device type outcomes (when log-transformed), linear regressions could not be used to compare smoking/vaping status user groups, or accurate vs. other relative harm perceptions, because the assumptions for linear regression were violated. Scores on the two device type variables did not significantly differ in either unadjusted or adjusted regression analyses between adults who smoked and vaped compared with adults who vaped and formerly smoked (Supplementary Table 2) or by accurate vs. other relative harm perceptions (Supplementary Table 3) (all p>.05).

Unadjusted and adjusted logistic regressions were used to compare smoking/vaping subgroups, then those with accurate vs. other relative harm perceptions, on dichotomised scores (0-5 [‘less/equal harm’] vs. 6-10 [‘more harm’]). There were no significant differences in these outcomes by smoking/vaping (Supplementary Table 4), or harm perceptions (Supplementary Table 5), except temperature to heat the e-liquid, which received a greater proportion of scores between 6 and 10 among those who had inaccurate relative harm perceptions of vaping (AOR=0.21, 0.05-0.93, p=.040) (Supplementary Table 5).

Adults who vaped and never regularly smoked, and adults who smoked but did not currently vape, could not be assessed in the regression models for most outcomes due to small cell counts (Supplementary Tables 2 and 4).

|  | **n** | **Log-transformed mean (SD)** | **Unadjusted** | |  | **Adjusted^1^** | |
| --- | --- | --- | --- | --- | --- | --- | --- |
| **Vaping product features by current smoking/vaping** |  |  | **Beta (95% CI)** | **p** |  | **Beta (95% CI)** | **p** |
| **Type of device (pod vs. tank), n=49** |  |  |  |  |  |  |  |
| a. Smoke and vape | 22 | 1.50 (0.53) | 0 |  |  | 0 |  |
| b. Vape, formerly smoked | 24 | 1.56 (0.44) | 0.05 (-0.23-0.34) | .712 |  | 0.02 (-0.26-0.3) | .879 |
| c. Vape, never regularly smoked | 2 | 1.45 (0.49) | - |  |  | - |  |
| d. Smoke, do not currently vape | 1 | 1.61 (.) | - |  |  | - |  |
| **Type of device (disposable vs. reusable), n=51** |  |  |  |  |  |  |  |
| a. Smoke and vape | 22 | 1.40 (0.71) | 0 |  |  | 0 |  |
| b. Vape, formerly smoked | 23 | 1.49 (0.54) | 0.09 (-0.26-0.45) | .605 |  | -0.02 (-0.36-0.31) | .888 |
| c. Vape, never regularly smoked | 2 | 1.59 (0.69) | - |  |  | - |  |
| d. Smoke, do not currently vape | 4 | 1.84 (0.29) | - |  |  | - |  |

**Supplementary Table 2. Linear regressions assessing the associations between vaping/smoking status groups and scores for the degree to which type of device (pod vs. tank; disposable vs. reusable) is** **perceived to contribute to the health harms of vaping.**

^1^ Adjusted for age group (18-29, 30-39, 40-49, 50+) and sex (male, female); - level of IV has n<10 participants providing a valid response to the DV and excluded via pairwise deletion.

|  | **n** | **Log-transformed mean (SD)** | **Unadjusted** | |  | **Adjusted^1^** | |
| --- | --- | --- | --- | --- | --- | --- | --- |
| **Vaping product features by harm perceptions** |  |  | **Beta (95% CI)** | **p** |  | **Beta (95% CI)** | **p** |
| **Type of device (pod vs. tank), n=49** |  |  |  |  |  |  |  |
| Accurate (vaping less harmful than smoking, ref) | 35 | 1.50 (0.47) | 0 |  |  | 0.00 |  |
| Other^2^ | 14 | 1.60 (0.49) | 0.1 (-0.2-0.4) | .498 |  | 0.16 (-0.18-0.5) | .347 |
| **Type of device (disposable vs. reusable), n=51** |  |  |  |  |  |  |  |
| Accurate (vaping less harmful than smoking, ref) | 37 | 1.54 (0.51) | 0 |  |  | 0.00 |  |
| Other^2^ | 14 | 1.66 (0.49) | -0.17 (-0.53-0.19) | .344 |  | -0.07 (-0.45-0.31) | .727 |

**Supplementary Table 3. Linear regressions assessing the associations between vaping harm perceptions and scores for the degree to which type of device (pod vs. tank; disposable vs. reusable) is** **perceived to contribute to the health harms of vaping.**

^1^ Adjusted for age group (18-29, 30-39, 40-49, 50+), sex (male, female), and smoking/vaping status (Smoke and vape, vape and formerly smoked, vape and never regularly smoked, smoke and do not currently vape); ^2^ ‘Other’ comprises perceptions that vaping is equally/more harmful than smoking, or don’t know/refused.

**Supplementary Table 4. Logistic regressions assessing the associations between vaping/smoking status groups high scores (>5) for the degree to which each feature is** **perceived to contribute to the health harms of vaping.**

| **Vaping product features by current smoking/vaping** | **Scores >5** |  | **Unadjusted** | |  | **Adjusted^1^** | |
| --- | --- | --- | --- | --- | --- | --- | --- |
|  | **% (n)** |  | **OR (95% CI)** | **p** |  | **aOR (95% CI)** | **p** |
| **Nicotine concentration, n=296** | **96.6 (286)** |  |  |  |  |  |  |
| a. Smoke and vape | 96.9 (125) |  | 1.00 |  |  | 1.00 |  |
| b. Vape, formerly smoked | 96.2 (101) |  | 0.81 (0.2-3.31) | .767 |  | 0.85 (0.21-3.52) | .823 |
| c. Vape, never regularly smoked | 100.0 (8) |  | - |  |  | - |  |
| d. Smoke, do not currently vape | 96.3 (52) |  | 0.83 (0.15-4.68) | .835 |  | 0.88 (0.15-4.97) | .881 |
| **Amount of e-liquid consumed, n=282** | **94 (265)** |  |  |  |  |  |  |
| a. Smoke and vape | 92.4 (110) |  | 1.00 |  |  | 1.00 |  |
| b. Vape, formerly smoked | 93.1 (95) |  | 1.11 (0.4-3.1) | .841 |  | 1.21 (0.43-3.44) | .717 |
| c. Vape, never regularly smoked | 100.0 (5) |  | - |  |  | - |  |
| d. Smoke, do not currently vape | 98.2 (55) |  | 4.5 (0.56-36.43) | .159 |  | 4.9 (0.6-40.13) | .138 |
| **Nicotine type (salt/freebase), n=88** | **65.9 (58)** |  |  |  |  |  |  |
| a. Smoke and vape | 57.8 (26) |  | 1.00 |  |  | 1.00 |  |
| b. Vape, formerly smoked | 71.0 (22) |  | 1.79 (0.67-4.74) | .244 |  | 1.8 (0.64-5.09) | .265 |
| c. Vape, never regularly smoked | 100.0 (1) |  | - |  |  | - |  |
| d. Smoke, do not currently vape | 81.8 (9) |  | 3.29 (0.64-16.99) | .155 |  | 3.32 (0.62-17.63) | .160 |
| **Temperature to heat the e-liquid, n=95** | **91.6 (87)** |  |  |  |  |  |  |
| a. Smoke and vape | 100.0 (41) |  | - |  |  | - |  |
| b. Vape, formerly smoked | 84.4 (27) |  | - |  |  | - |  |
| c. Vape, never regularly smoked | 100.0 (2) |  | - |  |  | - |  |
| d. Smoke, do not currently vape | 85.0 (17) |  | - |  |  | - |  |
| **Heat produced by device, n=92** | **85.9 (79)** |  |  |  |  |  |  |
| a. Smoke and vape | 82.9 (34) |  | 1.00 |  |  | 1.00 |  |
| b. Vape, formerly smoked | 84.4 (27) |  | 1.11 (0.32-3.9) | .868 |  | 1.06 (0.3-3.77) | .927 |
| c. Vape, never regularly smoked | 100.0 (1) |  | - |  |  | - |  |
| d. Smoke, do not currently vape | 94.4 (17) |  | 3.5 (0.4-30.8) | .259 |  | 3.62 (0.4-32.92) | .253 |
| **Amount of visible cloud of emissions, n=83** | **89.2 (74)** |  |  |  |  |  |  |
| a. Smoke and vape | 84.2 (32) |  | 1.00 |  |  | 1.00 |  |
| b. Vape, formerly smoked | 88.5 (23) |  | 1.44 (0.33-6.35) | .632 |  | 0.96 (0.3-3.15) | .952 |
| c. Vape, never regularly smoked | 100.0 (2) |  | - |  |  | - |  |
| d. Smoke, do not currently vape | 100.0 (17) |  | - |  |  | - |  |
| **Type of device (pod vs. tank), n=50** | **40**.0 **(20)** |  |  |  |  |  |  |
| a. Smoke and vape | 40.9 (9) |  | 1.00 |  |  | 1.00 |  |
| b. Vape, formerly smoked | 40.0 (10) |  | 0.96 (0.3-3.09) | .949 |  | 0.96 (0.3-3.15) | .952 |
| c. Vape, never regularly smoked | 50.0 (1) |  | - |  |  | - |  |
| d. Smoke, do not currently vape | 0.0 (0) |  | - |  |  | - |  |
| **Type of device (disposable vs. reusable), n=64** | **35.9 (23)** |  |  |  |  |  |  |
| a. Smoke and vape | 40.7 (11) |  | 1.00 |  |  | 1.00 |  |
| b. Vape, formerly smoked | 30.0 (9) |  | 0.62 (0.21-1.86) | .398 |  | 0.62 (0.2-1.88) | .398 |
| c. Vape, never regularly smoked | 50.0 (1) |  | - |  |  | - |  |
| d. Smoke, do not currently vape | 40.0 (2) |  | - |  |  | - |  |
| **Material of tank (e.g., glass, plastic), n=76** | **92.1 (70)** |  |  |  |  |  |  |
| a. Smoke and vape | 91.4 (32) |  | 1.00 |  |  | 1.00 |  |
| b. Vape, formerly smoked | 94.4 (17) |  | 1.59 (0.15-16.52) | .696 |  | 1.63 (0.15-17.34) | .686 |
| c. Vape, never regularly smoked | 100.0 (2) |  | - |  |  | - |  |
| d. Smoke, do not currently vape | 90.5 (19) |  | 0.89 (0.14-5.82) | .904 |  | 0.97 (0.14-6.69) | .976 |
| **Power/wattage of device, n=59** | **83.1 (49)** |  |  |  |  |  |  |
| a. Smoke and vape | 79.3 (23) |  | 1.00 |  |  | 1.00 |  |
| b. Vape, formerly smoked | 82.4 (14) |  | 1.22 (0.26-5.66) | .802 |  | 1.2 (0.26-5.62) | .813 |
| c. Vape, never regularly smoked | 100.0 (1) |  | - |  |  | - |  |
| d. Smoke, do not currently vape | 91.7 (11) |  | 2.87 (0.31-26.84) | .355 |  | 2.89 (0.31-27.09) | .354 |
| **Size of device, n=16** | **81.3 (13)** |  |  |  |  |  |  |
| a. Smoke and vape | 62.5 (5) |  | - |  |  | - |  |
| b. Vape, formerly smoked | 100.0 (4) |  | - |  |  | - |  |
| c. Vape, never regularly smoked | 0.0 (0) |  | - |  |  | - |  |
| d. Smoke, do not currently vape | 100.0 (4) |  | - |  |  | - |  |
| **Weight of device, n=9** | **55.6 (5)** |  |  |  |  |  |  |
| a. Smoke and vape | 66.7 (2) |  | - |  |  | - |  |
| b. Vape, formerly smoked | 25 (1) |  | - |  |  | - |  |
| c. Vape, never regularly smoked | 0.0 (0) |  | - |  |  | - |  |
| d. Smoke, do not currently vape | 100.0 (2) |  | - |  |  | - |  |

^1^ Adjusted for age group (18-39, 40+; dichotomised due to small cell sizes) and sex (male, female); - level of IV has n<10 participants providing a valid response to the DV and excluded via pairwise deletion.

**Supplementary Table 5. Logistic regressions assessing the associations between vaping harm perceptions and high scores (>5) for the degree to which each feature is** **perceived to contribute to the health harms of vaping.**

| **Vaping product features by harm perceptions** | **Scores >5** | |  | | **Unadjusted** | | |  | **Adjusted^1^** | | | |
| --- | --- | --- | --- | --- | --- | --- | --- | --- | --- | --- | --- | --- |
|  | **% (n)** |  | | **OR (95% CI)** | | **p** | |  | | **aOR^1^ (95% CI)** | **p** |  |
| **Nicotine concentration, n=296** | **96.6 (286)** |  | |  | | |  |  |  | |  | |
| Accurate (vaping less harmful than smoking, ref) | 96.6 (230) |  | | 1.00 | | |  |  | 1.00 | |  | |
| Other^2^ | 96.6 (56) |  | | 0.97 (0.2-4.71) | | | .974 |  | 0.9 (0.18-4.49) | | .898 | |
| **Amount of e-liquid consumed, n=282** | **94.0 (265)** |  | |  | | |  |  |  | |  | |
| Accurate (vaping less harmful than smoking, ref) | 93.3 (210) |  | | 1.00 | | |  |  | 1.00 | |  | |
| Other^2^ | 96.5 (55) |  | | 1.96 (0.44-8.85) | | | .379 |  | 2.42 (0.53-11.16) | | .257 | |
| **Nicotine type (salt/freebase), n=88** | **65.9 (58)** |  | |  | | |  |  |  | |  | |
| Accurate (vaping less harmful than smoking, ref) | 62.3 (43) |  | | 1.00 | | |  |  | 1.00 | |  | |
| Other^2^ | 78.6 (15) |  | | 2.27 (0.68-7.57) | | | .183 |  | 2.41 (0.69-8.46) | | .169 | |
| **Temperature to heat the e-liquid, n=95** | **91.6 (87)** |  | |  | | |  |  |  | |  | |
| Accurate (vaping less harmful than smoking, ref) | 94.7 (72) |  | | 1.00 | | |  |  |  | |  | |
| Other^2^ | 79.0 (15) |  | | **0.21 (0.05-0.93)** | | | **.040** |  | 0.24 (0.05-1.27) | | .093 | |
| **Heat produced by device, n=92** | **85.9 (79)** |  | |  | | |  |  |  | |  | |
| Accurate (vaping less harmful than smoking, ref) | 85.3 (58) |  | | 1.00 | | |  |  | 1.00 | |  | |
| Other^2^ | 87.5 (21) |  | | 1.21 (0.3-4.81) | | | .790 |  | 1.19 (0.27-5.22) | | .821 | |
| **Amount of visible cloud of emissions, n=83** | **89.2 (74)** |  | |  | | |  |  |  | |  | |
| Accurate (vaping less harmful than smoking, ref) | 87.5 (56) |  | | 1.00 | | |  |  | 1.00 | |  | |
| Other^2^ | 94.7 (18) |  | | 2.57 (0.3-21.98) | | | .388 |  | 1.94 (0.21-18.13) | | .560 | |
| **Type of device (pod vs. tank), n=50** | **40.0 (20)** |  | |  | | |  |  |  | |  | |
| Accurate (vaping less harmful than smoking, ref) | 41.7 (15) |  | | 1.00 | | |  |  | 1.00 | |  | |
| Other^2^ | 35.7 (5) |  | | 0.78 (0.22-2.79) | | | .700 |  | 0.62 (0.16-2.49) | | .506 | |
| **Type of device (disposable vs. reusable), n=64** | **35.9 (23)** |  | |  | | |  |  |  | |  | |
| Accurate (vaping less harmful than smoking, ref) | 37.0 (17) |  | | 1.00 | | |  |  | 1.00 | |  | |
| Other^2^ | 33.3 (6) |  | | 0.85 (0.27-2.69) | | | .786 |  | 0.93 (0.26-3.29) | | .906 | |
| **Material of tank (e.g., glass, plastic), n=76** | **92.1 (70)** |  | |  | | |  |  |  | |  | |
| Accurate (vaping less harmful than smoking, ref) | 93.1 (54) |  | | 1.00 | | |  |  | 1.00 | |  | |
| Other^2^ | 88.9 (16) |  | | 0.59 (0.1-3.54) | | | .566 |  | 0.7 (0.1-5.06) | | .723 | |
| **Power/wattage of device, n=59** | **83.1 (49)** |  | |  | | |  |  |  | |  | |
| Accurate (vaping less harmful than smoking, ref) | 85.4 (35) |  | | 1.00 | | |  |  | 1.00 | |  | |
| Other^2^ | 77.8 (14) |  | | 0.6 (0.15-2.45) | | | .477 |  | 0.56 (0.13-2.49) | | .450 | |
| **Size of device, n=16** | **81.3 (13)** |  | |  | | |  |  |  | |  | |
| Accurate (vaping less harmful than smoking, ref) | 77.8 (7) |  | | - | | |  |  | - | |  | |
| Other^2^ | 85.71 (6) |  | | - | | |  |  | - | |  | |
| **Weight of device, n=9** | **55.56 (5)** |  | |  | | |  |  |  | |  | |
| Accurate (vaping less harmful than smoking, ref) | 40.0 (2) |  | | - | | |  |  | - | |  | |
| Other^2^ | 75.0 (3) |  | | - | | |  |  | - | |  | |

^1^ Adjusted for age group (18-39, 40+; dichotomised due to small cell sizes), sex (male, female), and smoking/vaping status (Smoke and vape vs. otherwise; dichotomised due to small cell sizes); ^2^ ‘Other’ comprises perceptions that vaping is equally/more harmful than smoking, or don’t know/refused; - level of IV has n<10 participants providing a valid response to the DV and excluded via pairwise deletion.
