# Supplementary material for: Harm perceptions across vaping product features: An on‐line cross‐sectional survey of adults who smoke and/or vape in the United Kingdom
Source: Addiction. 2024 Jun 5;120(3):524–38. doi: 10.1111/add.16572 (PMC11813726; doi:10.1111/add.16572)
Supplement: Supplementary file 2 — Data S1. Supplementary Information. [file ADD-120-524-s002.pdf]

Perceptions of harms across vaping products: an online survey of adult smokers and vapers

---

Start of Block: Consent

<u>What is the purpose of the study?</u>

This survey is to help understand how people perceive the harms of using e-cigarettes or vaping products. It is conducted by Dr Katherine East at King's College London.

Before you decide whether you want to take part, it is important for you to understand why the research is being done and what your participation will involve. Please take time to read the following information carefully and discuss it with others if you wish. Ask me if there is anything that is not clear or if you would like more information.

<u>What will happen if I take part?</u>

If you agree to take part you will complete the survey anonymously. The survey will ask you questions about your perceptions of the harms of vaping as well as your experiences with vaping and smoking. To help protect your confidentiality, the survey will not contain information that will personally identify you. The survey will take you approximately 5 minutes to complete.

<u>Do I have to take part?</u>

Participation is completely voluntary. You should only take part if you want to and choosing not to take part will not disadvantage you in anyway. If you choose to take part you will be asked to provide your consent. To do this you will be asked to indicate that you have read and understand the information provided and that you consent to your anonymous data being used for the purposes explained.

You are free to withdraw at any point during completion of the survey, without having to give a reason. Withdrawing from the study will not affect you in any way. Once you submit the survey, it will no longer be possible to withdraw from the study because the data will be fully anonymous. Please do not include any personal identifiable information in your responses.

Data handling and confidentiality

This research is anonymous. This means that nobody, including the researchers, will be aware of your identity, and that nobody will be able to connect you to the answers you provide, even indirectly. Your answers will nevertheless be treated confidentially and the information you provide will not allow you to be identified in any research outputs/publications.

Your data will be held securely on Katherine East's King's College London OneDrive for Business account for a maximum of 10 years. A fully anonymised dataset with no identifiable data will also be uploaded to the Open Science Framework in January 2023 and made publicly available.

How is the project being funded?

This study is being funded by the Society for the Study of Addiction.

What will happen to the results of the study?

The results of the study will be summarised in academic publications and conference presentations.

Who should I contact for further information?

If you have any questions or require more information about this study, please contact me at katherine.east@kcl.ac.uk.

What if I have further questions, or if something goes wrong?

If this study has harmed you in any way or if you wish to make a complaint about the conduct of the study you can contact King's College London at rec@kcl.ac.uk.

Thank you for reading this information sheet and for considering taking part in this research.

If you give your consent to take part, please select "I agree" below.

○ I agree (1)

○ I do not agree (2)

Page Break

End of Block: Consent

Start of Block: Prolific ID

What is your Prolific ID?

Please note that this response should auto-fill with the correct ID. If it does not auto-fill, please manually provide your Prolific ID.

The research team will not be able to identify you without this.

____

End of Block: Prolific ID

Start of Block: Cig/ecig use

Do you regularly use electronic cigarettes and/or tobacco products (e.g. cigarettes, cigars)?

- ○ Regularly use both tobacco products and e-cigarettes (1)
- ○ Previously smoked tobacco products. Now only use e-cigarettes (2)
- ○ Only ever used e-cigarettes regularly (not tobacco products) (3)
- ○ Only use tobacco products (4)
- ○ Do not use e-cigarettes or tobacco products (5)
- ○ Rather not say (6)

End of Block: Cig/ecig use

Start of Block: Ecig and cig use detailed

Please select the response that best describes your experience with **vaping**:
By "vaping" we mean vaping e-cigarettes.

- ○ I currently use e-cigarettes (vape) daily (1)
- ○ I currently use e-cigarettes (vape) weekly but not daily (2)
- ○ I currently use e-cigarettes (vape) monthly but not weekly (3)
- ○ I have tried an e-cigarette but do not vape monthly (4)
- ○ I have never tried an e-cigarette (vape) (5)

Page Break

Please select the response that best describes your experience with **smoking**:
By “smoking” we mean smoking cigarettes, both factory-made and roll-your-own tobacco.

- ○ I currently use cigarettes (smoke) daily (1)
- ○ I currently use cigarettes (smoke) weekly but not daily (2)
- ○ I currently use cigarettes (smoke) monthly but not weekly (3)
- ○ I have tried a cigarette but do not smoke monthly (4)
- ○ I have never tried a cigarette (5)

Display This Question:

If Please select the response that best describes your experience with smoking:By “smoking” we mean... = I have tried a cigarette but do not smoke monthly

You said that you have tried a cigarette but do not smoke monthly. Please select the response that best describes your previous experience with **smoking**:

- ○ I previously used cigarettes (smoked) daily (1)
- ○ I previously used cigarettes (smoked) weekly but not daily (2)
- ○ I previously used cigarettes (smoked) monthly but not weekly (3)
- ○ I previously used cigarettes (smoked) less than monthly (4)
- ○ I have only ever tried a cigarette (5)

Display This Question:

If Please select the response that best describes your experience with smoking:By “smoking” we mean... = I have tried a cigarette but do not smoke monthly

When was the last time you smoked a cigarette?

- ○ Within the last 6 months (1)
- ○ Not in the past 6 months but some time in the past 12 months (2)
- ○ 1 to 4 years ago (3)
- ○ 5 or more years ago (4)
- ○ Don't know (5)

End of Block: Ecig and cig use detailed

Start of Block: ITC harm perception measures

Compared to smoking cigarettes, how harmful do you think using e-cigarettes/vaping is?

- ○ Vaping is much less harmful than smoking cigarettes (1)
- ○ Vaping is somewhat less harmful than smoking cigarettes (2)
- ○ Vaping is equally harmful to smoking cigarettes (3)
- ○ Vaping is somewhat more harmful than smoking cigarettes (4)
- ○ Vaping is much more harmful than smoking cigarettes (5)
- ○ Don't know (6)

Page Break

Are you worried -- or would you be worried if you vaped -- that using e-cigarettes/vaping will damage your health in the future?

- Not at all worried (1)
- A little worried (2)
- Moderately worried (3)
- Very worried (4)
- Don’t know (5)

End of Block: ITC harm perception measures

Start of Block: Harm perceptions block 1

These questions are about vaping product features and any effects on the **health** of the **user**.

Which of the following features do you believe might have any effect on the health harms of vaping?

Select all that apply.

There will be an opportunity to explain your answer at the bottom of this page.

- ☐ Amount of e-liquid consumed (1)
- ☐ Amount of visible cloud (or plume) of emissions (2)
- ☐ Nicotine concentration (4)
- ☐ Nicotine type (e.g., salt or freebase) (5)
- ☐ Heat produced by the device (i.e., how hot to the touch the device is) (19)
- ☐ Temperature to heat the e-liquid (3)
- ☐ Power/wattage of the device (6)
- ☐ Flavours (e.g., tobacco, menthol, fruit) (7)
- ☐ Type of device (e.g., disposable, pod, tank) (8)
- ☐ Material of the tank (e.g., glass, plastic) (9)
- ☐ Where the product is sourced or purchased (e.g., vape shop, online) (10)
- ☐ Weight of the device (11)
- ☐ Size of the device (12)
- ☐ Brand of the device (13)
- ☐ Brand of the e-liquid (14)

☐ Other (please specify) (15)

☐ ⊗None of the above (18)

Would you like to explain your answer to the above question? If yes, please do so here:

○ No (1)

○ Yes (please explain) (2) ____

End of Block: Harm perceptions block 1

Start of Block: Harm perceptions block 2

Below are the features that you believed might influence how harmful a vaping product is to the **health** of the **user**.
Please indicate where along the scale you perceive the greatest harm to users' health.
There will be an opportunity to explain your answer at the bottom of this page.

Amount of e-liquid consumed (less vs. more)

	Less e-liquid consumed is more harmful	More e-liquid consumed is more harmful	Don't know
	0 1 2 3 4 5 6 7 8 9 10		
()			

Display This Question:

If These questions are about vaping product features and any effects on the health of the user. Whic... = Amount of visible cloud (or plume) of emissions

Amount of visible cloud (or plume) of emissions (small vs. large)

	Small visible cloud of emissions is more harmful	Large visible cloud of emissions is more harmful	Don't know
	0 1 2 3	4 5 6 7	8 9 10
()			

Display This Question:

If These questions are about vaping product features and any effects on the health of the user. Whic... = Temperature to heat the e-liquid

Heat produced by the device (i.e., how hot to the touch the device is; less vs. more)

	Less heat is more harmful	More heat is more harmful	Don't know
	0 1 2 3	4 5 6 7	8 9 10
()			

Display This Question:

If These questions are about vaping product features and any effects on the health of the user. Whic... = Temperature to heat the e-liquid

Temperature to heat the e-liquid (less vs. more)

	Lower temperature is more harmful	Higher temperature is more harmful	Don't know
	0 1 2 3	4 5 6 7	8 9 10
()			

Display This Question:

If These questions are about vaping product features and any effects on the health of the user. Whic... = Nicotine concentration

Nicotine concentration (0% vs. 2%)

	0% nicotine (or 0mg/mL) is more harmful	2% nicotine (or 20mg/mL) is more harmful	Don't know
	0 1 2 3 4 5 6 7 8 9 10		
()			

Display This Question:

If These questions are about vaping product features and any effects on the health of the user. Whic... = Nicotine type (e.g., salt or freebase)

Nicotine type (freebase vs. salt)

	Don't know
	0 1 2 3 4 5 6 7 8 9 10
()	

Display This Question:

If These questions are about vaping product features and any effects on the health of the user. Whic... = Power/wattage of the device

Device power (low vs. high)

	Low device power is more harmful	High device power is more harmful	Don't know
	0 1 2 3 4 5 6 7 8 9 10		
()			

Display This Question:

If These questions are about vaping product features and any effects on the health of the user. Whic... = Type of device (e.g., disposable, pod, tank)

Device type (disposable vs. refillable)

	Disposable device is more harmful	Reusable device is more harmful	Don't know

0 1 2 3 4 5 6 7 8 9 10

()

Display This Question:

If These questions are about vaping product features and any effects on the health of the user. Whic... = Type of device (e.g., disposable, pod, tank)

Device type (pod vs. tank)

	Pod device is more harmful	Tank device is more harmful	Don't know

0 1 2 3 4 5 6 7 8 9 10

()

Display This Question:

If These questions are about vaping product features and any effects on the health of the user. Whic... = Material of the tank (e.g., glass, plastic)

Material of the tank (glass vs. plastic)

	Glass tank is more harmful	Plastic tank is more harmful	Don't know

0 1 2 3 4 5 6 7 8 9 10

()	

Display This Question:

If These questions are about vaping product features and any effects on the health of the user. Whic... = Weight of the device

Weight of the device (light vs. heavy)

	Lighter device is more harmful					Heavier device is more harmful				Don't know	
	0	1	2	3	4	5	6	7	8	9	10
()											

Display This Question:

If These questions are about vaping product features and any effects on the health of the user. Whic... = Size of the device

Size of the device (small vs. large)

	Smaller device is more harmful					Larger device is more harmful				Don't know	
	0	1	2	3	4	5	6	7	8	9	10
()											

Display This Question:

If These questions are about vaping product features and any effects on the health of the user. Whic... = Flavours (e.g., tobacco, menthol, fruit)

Please state which flavour(s) you perceive to be the most, and least, harmful.

☐ Most harmful flavour(s): (1) ____

☐ Least harmful flavour(s): (2) ____

Display This Question:

If These questions are about vaping product features and any effects on the health of the user. Whic... = Where the product is sourced or purchased (e.g., vape shop, online)

Please state which purchase sources or locations you perceive to be the most, and least, harmful.

☐ Most harmful source(s) or location(s): (1) ____

☐ Least harmful source(s) or location(s): (2) ____

Display This Question:

If These questions are about vaping product features and any effects on the health of the user. Whic... = Brand of the device

And These questions are about vaping product features and any effects on the health of the user. Whic... = Brand of the e-liquid

Please state which brand(s) you perceive to be the most, and least, harmful.

☐ Most harmful brand(s): (1) ____

☐ Least harmful brand(s): (2) ____

Would you like to explain any of your answers to the above questions? If yes, please do so here:

- ○ No (1)
- ○ Yes (please explain) (2) ____

End of Block: Harm perceptions block 2
